# Combination treatment of an IDH1 inhibitor with chemotherapy in IDH1 mutant acute myeloid leukemia

**DOI:** 10.1007/s00277-020-04001-w

**Published:** 2020-04-15

**Authors:** Charu Gupta, Stefan Kaulfuss, Kerstin Görlich, Basem Othman, Anuhar Chaturvedi, Michael Heuser

**Affiliations:** 1 Department of Hematology, Hemostasis, Oncology and Stem Cell Transplantation, Hannover Medical School, Hannover, Germany; 2 Bayer AG, Berlin, Germany

Dear Editor,

The first clinical IDH1 inhibitor ivosidenib as a single agent in *IDH1*-mutated relapsed or refractory acute myeloid leukemia (AML) showed an overall response rate of 41.6% and a complete remission rate of 21.6% with a median duration of response of 8.2 months [1]. While these results are promising in this difficult to treat patient setting, they also suggest that mIDH1 inhibitors should be combined with other agents to improve efficacy. *IDH1* mutations do not show a clear prognostic effect in AML patients who are treated with standard induction and consolidation therapy [2–5]. It is unclear how an IDH1 inhibitor acts in combination with standard chemotherapy and how the treatment sequence may affect treatment efficacy.

We evaluated the mIDH1 inhibitor BAY1436032 in sequential or simultaneous combination with cytarabine plus doxorubicin in a previously reported IDH1 mutant PDX mouse model [6] (Fig. 1a). All treatment groups that were treated with BAY1436032 received the drug for 87 days (Fig. 1a). While the engraftment of human leukemic cells increased in the vehicle-treated mice at week 8 and in chemotherapy-treated mice at week 12 after the start of treatment, the percentage of leukemic cells decreased in BAY1436032-treated mice as well as in the groups receiving the sequential and simultaneous combination treatments (Fig. 1b). However, after the stop of treatment at week 12, the percentage of leukemic cells increased after week 16 in the group receiving BAY1436032 and after week 24 in the group treated with a sequential combination of BAY1436032 and chemotherapy (Fig. 1b). Similar to the combination with azacitidine [7], the percentage of leukemic cells in mice treated with the simultaneous combination of BAY1436032 and chemotherapy showed a delayed increase of blasts and slower leukemia kinetics (Fig. 1b). Importantly, 4 of 8 mice from this cohort had less than 10% human leukemic cells in the peripheral blood at the end of the study at 48 weeks (Fig. 1c). WBC counts constantly increased and hemoglobin as well as platelet counts decreased in all treatment groups but stayed normal in the group of mice treated simultaneously with BAY1436032 and chemotherapy (Fig. 2a, b, and c). While chemotherapy-treated mice survived longer with a median survival of 206 days compared with vehicle-treated mice with a median survival of 173 days, BAY1436032-treated mice had significantly longer latency with a median survival of 325 days. However, no significant difference in survival was observed between mice treated with BAY1436032 alone and mice treated sequentially with the combination of BAY1436032 and chemotherapy (median survival of 340 days). Importantly, 5/8 mice treated simultaneously with BAY1436032 and chemotherapy survived until the end of the study at 400 days and the median survival was not reached (Fig. 2d). In summary, only the simultaneous combination of BAY1436032 and chemotherapy showed additive effects in IDH1-mutated human leukemia in vivo.

**Fig. 1:**
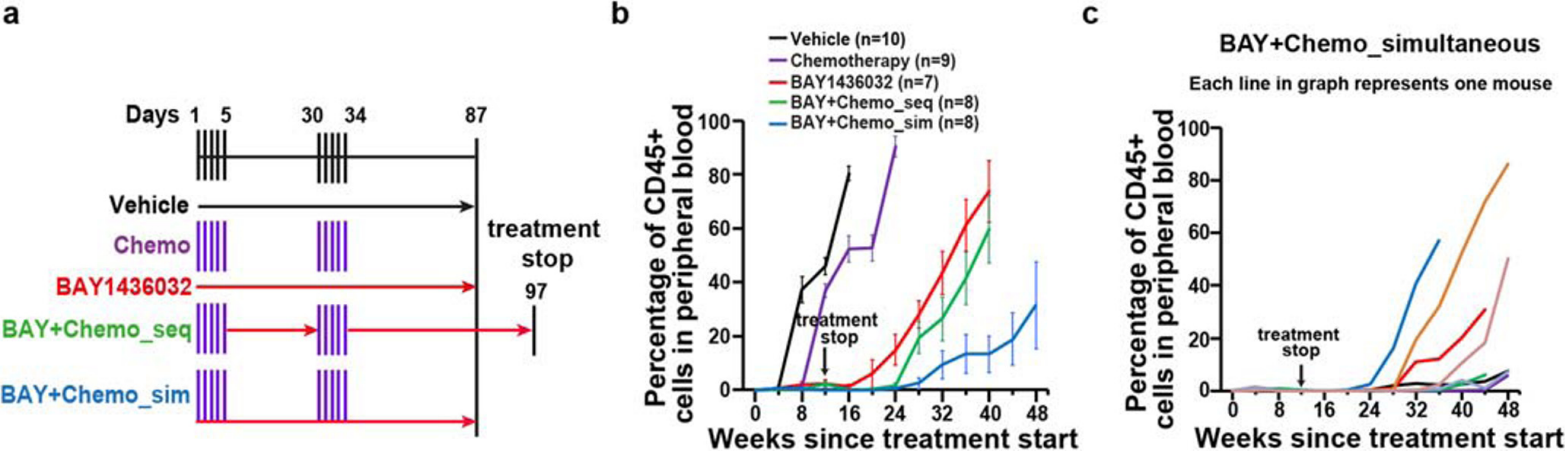
Mutant IDH1 inhibitor BAY1436032 combined with chemotherapy delays engraftment of leukemic cells in a patient-derived IDH1 mutant AML xenograft model in vivo. **a** Schematic representation of the treatment regimens; sim, simultaneous treatment with BAY1436032 and chemotherapy; seq, sequential treatment with BAY1436032 and chemotherapy. **b** Percentage of hCD45+ leukemic cells in peripheral blood of IDH1mutant (R132C) PDX mice at different time points after treatment start with vehicle, chemotherapy (cytarabine 50 mg/kg plus doxorubicin 1 mg/kg, days 1–5 and days 30–34), BAY1436032 (150 mg/kg, p.o., continuously), or the sequential or simultaneous combination of BAY1436032 and chemotherapy according to the treatment regimen shown in Fig. 1a (mean ± SEM). **c** Percentage of hCD45+ leukemic cells in peripheral blood of individual mice transplanted with human IDH1 mutant AML cells and simultaneously treated with BAY1436032 and chemotherapy

**Fig. 2:**
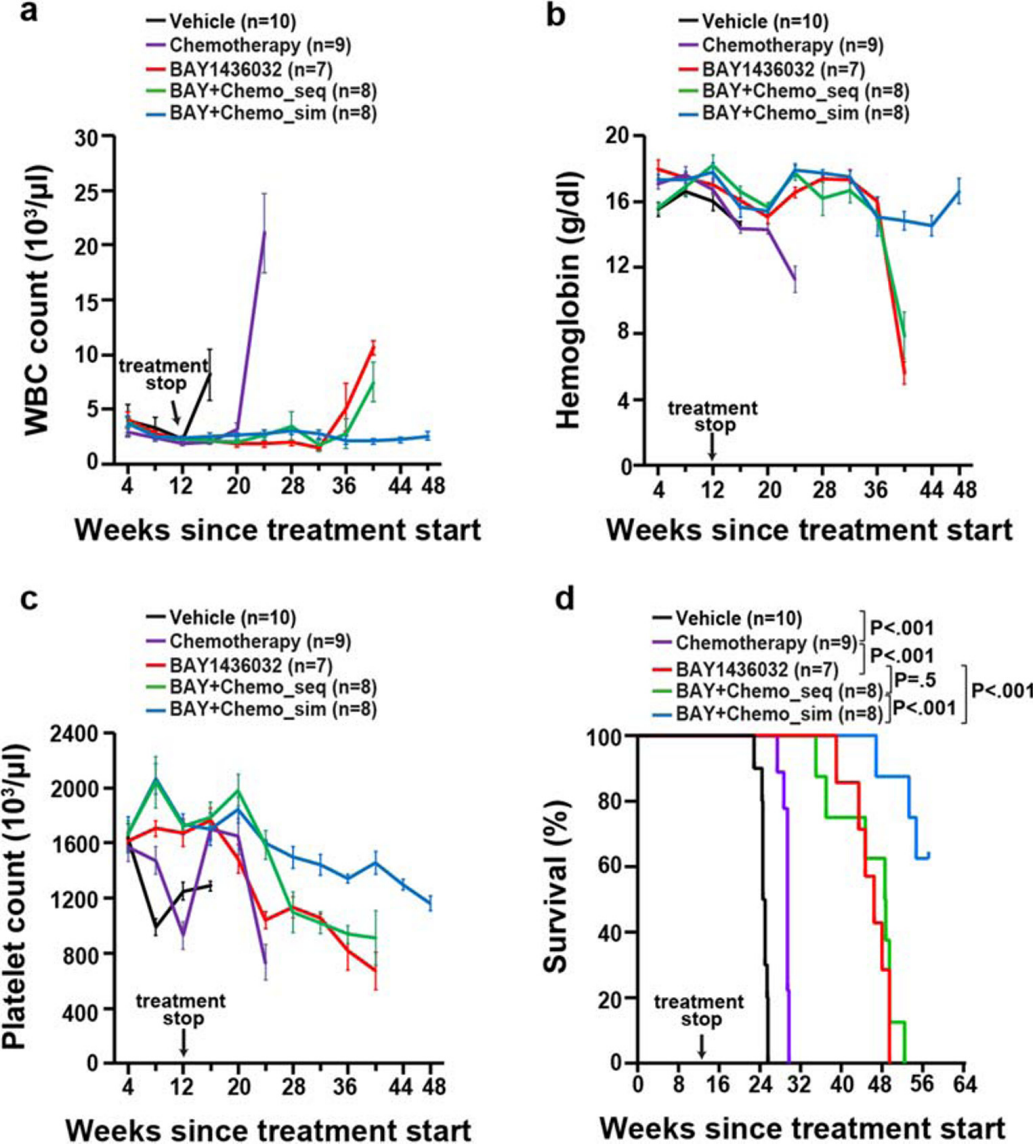
Mutant IDH1 inhibitor BAY1436032 combined with chemotherapy improves survival when simultaneously applied to an AML PDX model. **a** White blood cell counts after different time points after treatment start with vehicle, chemotherapy (cytarabine 50 mg/kg plus doxorubicin 1 mg/kg, days 1–5 and days 30–34), BAY1436032 (150 mg/kg, p.o., q.d., continuously), or the sequential or simultaneous combination of BAY1436032 and chemotherapy according to the treatment regimen shown in Fig. 1a (mean ± SEM). **b** Hemoglobin after different time points after the start of treatment (mean ± SEM). **c** Platelet count in the peripheral blood of IDH1 mutant PDX mice at different time points after the start of treatment (mean ± SEM). **d** Kaplan–Meier survival curves of IDH1mutant PDX mice treated with vehicle, chemotherapy, BAY1436032, or the sequential or simultaneous combination of BAY1436032 and chemotherapy according to the treatment regimen shown in Fig. 1a

The findings from our preclinical study show that simultaneously combining an IDH1mutant inhibitor with cytarabine plus doxorubicin substantially inhibits leukemia in vivo. These findings are in accordance with our previous study in which we showed a synergistic effect of simultaneous administration of an IDH1 inhibitor with the hypomethylating agent azacitidine compared with sequential administration [7]. A phase 1 study of a mutant IDH1 inhibitor plus chemotherapy in newly diagnosed IDH1mut AML and MDS patients has been initiated (NCT03839771). Initial data have shown a response rate (CR, CRi, or CRp) of 93% in patients with de novo AML and 63% in patients with secondary AML [8]. Our data strongly argues for the concurrent application of mIDH1 inhibitors with chemotherapy, and thus informs the design of future studies and predicts improved outcome of this regimen in IDH1-mutated AML patients.

## Electronic supplementary material

ESM 1 (DOCX 22 kb)

## Abbreviations

IDH1: isocitrate dehydrogenase 1
mIDH1: mutant isocitrate dehydrogenase 1
CR: complete remission
CRi: CR with incomplete hematologic recovery
CRp: CR with incomplete platelet counts
sAML: secondary acute myeloid leukemia
