# Supplementary material for: Combination treatment of an IDH1 inhibitor with chemotherapy in IDH1 mutant acute myeloid leukemia
Source: Ann Hematol. 2020 Apr 15;99(6):1415–7. doi: 10.1007/s00277-020-04001-w (PMC7237524; doi:10.1007/s00277-020-04001-w)
Supplement: Supplementary file 1 — (DOCX 22 kb) [file 277_2020_4001_MOESM1_ESM.docx]

**Combination Treatment of an IDH1 Inhibitor with Chemotherapy in IDH1 mutant Acute Myeloid Leukemia**

Charu Gupta,^1^ Stefan Kaulfuss,^2^ Kerstin Görlich,^1^ Basem Othman,^1^ Anuhar Chaturvedi^1*^ and Michael Heuser^1*^

**Supplementary Methods**

**Transplantation and treatment**

6-8 weeks old female NOD.Cg-*Prkdc^scid^ Il2rg^tm1Wjl^*/SzJ (NSG) mice were bred at Hannover Medical School, Germany and kept in pathogen free conditions at the central animal laboratory of Hannover Medical School. Experimental procedures were approved by governmental authorities of Lower Saxony, Germany, and supervised by local animal welfare officials. The IDH1mut AML PDX model was developed as described.[[8](#_ENREF_8)] One million patient-derived AML cells (hCD45+) were collected from bone marrow and spleen of leukemic mice and were injected intravenously in the tail vein of sublethally (3 Gy) irradiated NSG mice. Neither randomization nor blinding was used since all animal experiments were performed with a homogeneous strain, at comparable age of the mice. Treatment was started 28 days after transplantation. The control groups were treated with either vehicle, BAY (150 mg/kg once daily p.o. continuously), or chemotherapy, which consisted of cytarabine (50 mg/kg once daily days 1-5 i.v.) and doxorubicin (1 mg/kg once daily days 1-3 i.v.). The treatment was repeated once after 29 days. The test groups were treated with BAY and chemotherapy in the doses mentioned above either starting both drugs on day 1 (simultaneous group) or starting chemotherapy on day 1 and BAY on day 6 (sequential group). Treatment with BAY was stopped after 12 weeks. The proportion of leukemic cells in peripheral blood of mice was measured with the human-specific CD45 antibody every four weeks by tail vein bleeds and flow cytometry.[[9](#_ENREF_9)] Blood counts were performed using an ABC Vet Automated Blood counter (Scil animal care company GmbH, Viernheim, Germany). All animal experiments were started with 10 animals, however, the animals which died before the start of treatment due to engraftment failure, during injections or during bleeding were excluded in the study.
